# Moderated mediation with composites: The composite moderated structural equations approach

**DOI:** 10.3758/s13428-025-02930-w

**Published:** 2026-04-06

**Authors:** Tamara Schamberger, Florian Schuberth, Jörg Henseler

**Affiliations:** 1 Faculty of Business Administration and Management, Bielefeld University, Universitätsstraße 25, Bielefeld, 33615 Germany; 2 Department of Design, Production and Management, University of Twente, Enschede, 7500 AE The Netherlands; 3 Nova Information Management School, Campus de Campolide, Universidade Nova de Lisboa, 1070-312 Lisboa, Portugal

**Keywords:** Composites, Mediation, Moderation, Latent moderated structural equations, Henseler–Ogasawara specification, Formative constructs

## Abstract

Moderated mediation models are crucial in many disciplines, particularly the social sciences. Researchers use them to analyze the conditions under which different variables are related. Structural equation modeling (SEM) is an eminently suitable framework for this endeavor. In fact, several approaches have been proposed and extended to model moderated mediation effects involving reflectively measured latent variables. However, approaches to modeling moderated mediation involving unknown-weight composites (i.e., weighted linear combinations of variables whose weights are estimated freely) are limited in either model specification or model assessment. Unknown-weight composites are used, for example, to model formative constructs or collections of heterogeneous causes. In this study, we propose composite moderated structural equations (CMS), a new approach that combines latent moderated structural equations (LMS), the standard SEM approach for estimating moderation effects among latent variables, with the H–O specification, a recently introduced specification for flexibly modeling composites. A Monte Carlo simulation demonstrates the performance of CMS and confirms that CMS enables researchers to flexibly model and estimate moderated mediation effects involving unknown-weight composites.

**Supplementary Information:**

The online version contains supplementary material available at 10.3758/s13428-025-02930-w.

## Introduction

Researchers in the social and behavioral sciences frequently study constructs and their relations (Bollen, 1989). Often, the conditions under which constructs are related, i.e., mediation, moderation, and moderated mediation effects, are of interest (Little, Card, Bovaird, Preacher, & Crandall, 2007). A mediator variable transmits the effect of one variable onto another variable, while a moderator variable influences the effect between two variables (Baron & Kenny, 1986). A promising approach to studying the relationship between constructs is structural equation modeling (SEM, Bollen, 1989; Bollen & Paxton, 1998; Gunzler, Chen, Wu, & Zhang, 2013). In SEM, constructs are traditionally modeled as latent variables, particularly, as common factors that explain the variance–covariance structure of their related observed variables (Bollen, 1989; Bollen & Lennox, 1991). Various approaches have been developed to study moderation effects between latent variables. Today, the latent moderated structural equations (LMS) approach (Klein & Moosbrugger, 2000) is arguably the standard approach for studying nonlinear structural equation models with reflectively measured latent variables (Dijkstra & Schermelleh-Engel, 2014; Ng & Chan, 2020). Additionally, the quasi-maximum likelihood approach (Klein & Muthén, 2007) and various product-indicator approaches (see e.g., Kenny, & Judd, 1984; Marsh, Wen, & Hau, 2004; Wall & Amemiya, 2001) have been introduced to study moderation effects in structural equation models. These approaches have subsequently been extended to study moderated mediation models (Slupphaug, Mehmetoglu, & Mittner, 2025; see also Feng, Song, Zhang, Zheng, & Pan, 2020).

Not all constructs studied in the social sciences should be modeled as latent variables (Bollen, 2011a; Bollen & Bauldry, 2011b; Edwards & Bagozzi, 2000; Schuberth, 2023; Yu, Zaza, Schuberth, & Henseler, 2021). For example, a theoretical construct representing a collection of heterogeneous causes is better modeled as a composite, i.e., a linear combination of variables (Grace & Bollen, 2008; Yu, Schuberth, & Henseler, 2025). Similarly, constructs representing indices, treatments, skills, or activities should be modeled as composites because they emerge from other variables (Alamer, Schuberth, & Henseler, 2024; Henseler, 2021; Schamberger, Schuberth, & Henseler, 2023).

Although composites are frequently encountered in the social sciences, studying them in the context of moderated mediation is challenging because the SEM literature lacks established approaches. One way to model unknown-weight and fixed-weight composites in SEM is the one-step approach (Grace & Bollen, 2008), whereby a composite is modeled as a formatively measured latent variable whose error term’s variance is fixed to zero. Using this approach to include a composite in a moderated mediation model resembles modeling the composite as a latent variable in a multiple indicator multiple cause (MIMIC) model (Jöreskog & Goldberger, 1975). However, this approach has limited flexibility for modeling composites (e.g., Schuberth, 2023), which is particularly problematic in the context of moderated mediation as it does not permit modeling a composite as a variable affected by other variables, such as a mediator. Other SEM approaches, such as the two-step approach or pseudo-indicator approach (Rose, Wagner, Mayer, & Nagengast, 2019) are limited to fixed-weight composites, i.e., composites whose weights were determined prior to the analysis. Against this background, this paper addresses the following question: How can unknown-weight composites be studied in moderated mediation models using SEM?

To answer this question, we introduce composite moderated structural equations (CMS), which combines LMS with the recently introduced H–O specification (named after their main contributors, Henseler and Ogasawara, see Schuberth, 2023) to flexibly model composites in SEM. As a result, CMS allows researchers to study composites as moderated mediators in SEM.

The remainder of our paper is organized as follows. In the next section, we discuss moderated mediation in SEM. Specifically, we explain what moderated mediation is and provide a brief overview of existing approaches to studying the moderated mediation effects of reflectively measured latent variables. In addition, we discuss various approaches to studying unknown-weight composites in SEM, focusing particularly on their potential for studying moderated mediation involving composites. Next, we introduce the CMS approach, which combines LMS and the H–O specification. Subsequently, we demonstrate CMS’s performance using a Monte Carlo simulation. The paper concludes with a discussion of the benefits and limitations of our approach.

**Fig. 1:**
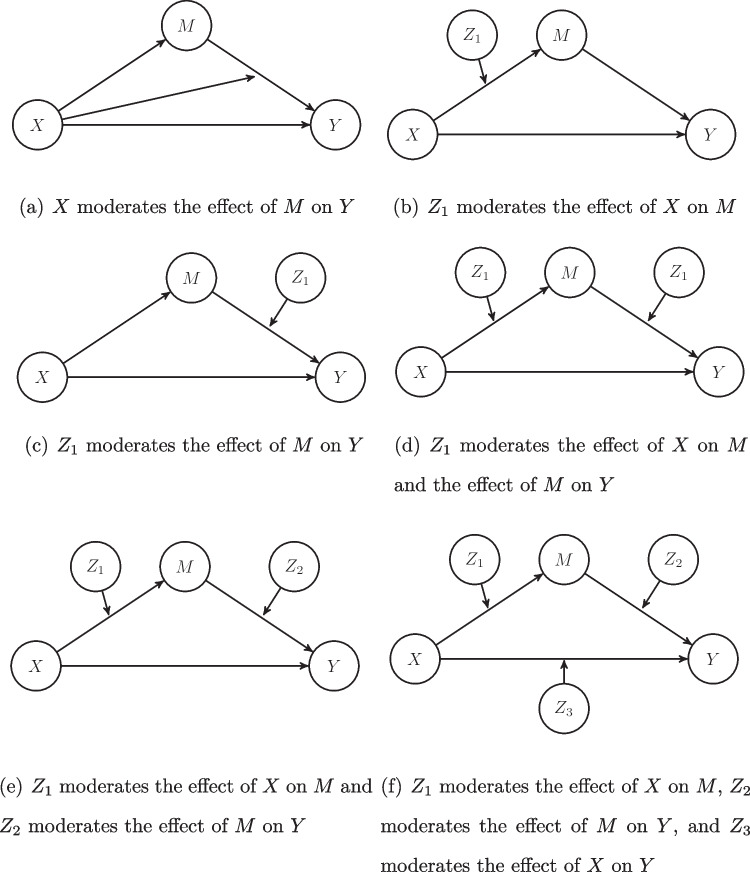
Different types of moderated mediation

## Moderated mediation in SEM

Social and behavioral scientists often use SEM to study moderated mediation effects (Little et al., 2007; Ng & Chan, 2020). However, existing SEM approaches for studying moderated mediation have mainly been developed for observed and latent variables. These approaches are limited when it comes to composites. In the following subsection, we provide a brief explanation of moderated mediation. Subsequently, we present SEM approaches for studying the moderated mediation effects of reflectively measured latent variables. The section concludes with a discussion of existing SEM approaches for studying composites and their potential for studying unknown-weight composites as moderated mediators.

### Mediation + moderation = moderated mediation

Moderated mediation is a combination of mediation and moderation (Preacher, Rucker, & Hayes, 2007). It is used in various fields such as early intervention research (Hopwood, 2007), sociology (Wang & Wu, 2023), economics (Li, Dai, & Cui, 2020), psychology (Liu, Ni, & Niu, 2021), organizational research (Sardeshmukh & Vandenberg, 2017), and behavioral research (Okazaki, Schuberth, Tagashira, & Andrade, 2021).

In the linear *mediation* model, the predictor variable *X* causes the mediator variable *M*, which in turn causes the outcome variable *Y*. In other words, *X* has an indirect effect on *Y* through the mediator *M*. For mediation with a single mediator, three different types of mediation can be distinguished (Zhao, Lynch, & Chen, 2010).

- *Full mediation*: The predictor variable *X* affects *Y* only indirectly through the mediator *M*, which means that *X* has no direct effect on *Y*.
- *Complementary partial mediation*: The predictor variable *X* has a direct and indirect effect on *Y*, and these effects are in the same direction.
- *Competitive partial mediation*: The predictor variable *X* has a direct and an indirect effect on *Y*, but the two effects are in different directions.

Guidelines for testing for mediation effects can be found in Little et al. (2007) and Zhao et al. (2010).

In addition to mediation, researchers often study *moderation* (Baron & Kenny, 1986; Cohen, Cohen, West, & Aiken, 2013). Moderation occurs when the strength of the effect of *X* on *Y* depends on the level of a third variable *Z*, i.e., the moderator (Preacher et al., 2007). Thus, the moderator *Z* interacts with the predictor *X* to explain the outcome variable *Y*. Therefore, a moderation effect is also called an *interaction effect* (Hayes, 2017). Guidelines on how to study moderation effects can be found in Hayes (2017).

Moderation and mediation can also occur together. This is known as *moderated mediation*. “[M]oderated mediation occurs when the strength of an indirect effect depends on the level of some variable” (Preacher et al., 2007, p. 193). As shown in Fig. 1, different cases of moderated mediation can be distinguished given a variable *X*, a mediator *M*, an outcome variable *Y*, and two other moderator variables, $Z_1$ and $Z_2$ (Preacher et al., 2007).

### SEM approaches to study moderated mediation

SEM is particularly suitable for studying moderated mediation. Unlike classical regression analysis, SEM allows the specification of a set of equations, which is necessary for studying mediation effects. Additionally, SEM enables the specification of moderation effects. In principle, SEM can emulate all procedures based on the general linear model (Graham, 2008). Furthermore, it can be used to study the relations between constructs. Arguably, constructs in SEM are most often modeled as latent variables that are measured reflectively (Rhemtulla, van Bork, & Borsboom, 2020). These types of constructs are also referred to as *reflective constructs* (Petter, Straub, & Rai, 2007). Several classes of approaches have been developed to study moderation effects involving reflectively measured latent variables. Product-indicator approaches include the classical product-indicator approach (Kenny & Judd, 1984), the generalized appended product-indicator approach (Wall & Amemiya, 2001), the orthogonalizing approach (Little, Bovaird, & Widaman, 2006), and the unconstrained product-indicator approach (Marsh et al., 2004). Two-stage or factor-score-based approaches comprise the two-stage least-squares approach (Bollen, 1995) and the two-stage method-of-moments approach (Ng & Chan, 2020; Wall & Amemiya, 2000, 2003). Distribution-analytic approaches cover the quasi maximum likelihood approach (Klein & Muthén, 2007), the marginal maximum likelihood approach (Jin, Vegelius, & Yang-Wallentin, 2020), and the latent moderated structural equations (LMS) approach (Klein & Moosbrugger, 2000). Finally, Bayesian approaches (Kelava & Nagengast, 2012; Lee & Song, 2004), the single-indicator approach (Ping, 1995), the structural-after-measurement approach (Rosseel, Burghgraeve, Loh, & Schermelleh-Engel, 2025a), and consistent partial least squares path modeling (Dijkstra & Schermelleh-Engel, 2014) provide further alternatives. Some approaches can be applied directly to moderated mediation models (e.g., Chen, 2015; Cheung & Lau, 2015; Feng et al., 2020; Irmer, Klein, & Schermelleh-Engel, 2024). Others, such as QML, have been extended to this context (Slupphaug et al., 2025).

In addition to reflectively measured latent variables, researchers are also concerned with formative constructs. While reflectively measured latent variables are assumed to be the underlying cause of their measures (Fornell & Bookstein, 1982), formative constructs are formed by a set of observed variables (Petter et al., 2007). A formative construct in SEM can be modeled in at least two ways. First, it can be considered as a latent variable in a causal-formative measurement model (e.g., Jarvis, MacKenzie, & Podsakoff, 2003; Petter et al., 2007). In this case, the observed variables are considered as causes of the latent variable (Blalock, 1964; Bollen, 1984; Bollen & Diamantopoulos, 2017; Bollen & Lennox, 1991; Jöreskog & Goldberger, 1975; Rindskopf, 1984) and are therefore also referred to as *causal indicators* (Bollen & Bauldry, 2011b). Since it is expected that not all variation in the latent variable is explained by the causal indicators, an error term is added at the construct level. Second, it has been proposed to model a formative construct as a composite (e.g., Cho, Hwang, & Sarstedt, 2022; Fornell, 1982; Henseler & Schuberth, 2021). In this case, the construct is a (weighted) linear combination of its observed variables, which are referred to as *components*. In contrast to the causal-formative measurement model, no error term is added at the construct level, so the construct is assumed to be fully composed of its observed variables. To emphasize the difference from latent variables, Cole, Maxwell, Arvey, and Salas (1993) suggested talking about *emergent variables* (see also Henseler, 2021).

Contrary to common belief, it has been shown that the meaning of the latent variable in the causal-formative measurement model is not determined by its causal indicators. Specifically, it has been shown that the relations from the latent variable in the causal-formative measurement model to other variables do not change when causal indicators are omitted. “If causal indicators can be omitted without any consequences to parameter estimates or interpretation of the structural part of the model, then a latent variable with causal indicators cannot possibly derive its meaning from the causal indicators themselves” (Aguirre-Urreta, Rönkkö, & Marakas, 2016a, p. 93). Consequently, the causal-formative measurement model is not consistent with our understanding of formative constructs: Formative constructs are composed of their observed variables (Bagozzi & Phillips, 1982; Fornell & Bookstein,^1982^; MacKenzie, Podsakoff, & Jarvis, 2005), so the meaning of a formative construct should change when an observed variable is removed (Jarvis et al., 2003). For a critical discussion of the causal-formative measurement model, the interested reader is referred to Howell, Breivik, and Wilcox (2007a, b); Bollen (2007); Bagozzi (2007); Bainter and Bollen (2014), including the various commentaries on their original article; Aguirre-Urreta, Rönkkö, & Marakas (2016a, b); Bollen and Diamantopoulos (2017); Cadogan and Lee (2016); Lee and Chamberlain (2016). Against this background, we focus on modeling formative constructs as composites.

### Approaches to moderated mediation with formative constructs

One approach to studying moderated mediation with formative constructs is partial least squares path modeling (PLS-PM; Henseler & Chin, 2010; Wold, 1982). Since PLS-PM only produces consistent parameter estimates when all constructs are modeled as composites (Dijkstra, 2017), consistent partial least squares (PLSc) was introduced (Dijkstra & Henseler, 2015a, b; Rademaker & Schuberth, 2020). PLSc can consistently estimate models containing both latent variables and composites (Schuberth, Henseler, & Dijkstra, 2018b). To estimate the parameters of models with moderated mediation effects, PLSc performs three steps (Dijkstra & Schermelleh-Engel, 2014; Schuberth, 2019). In the first step, the partial least squares algorithm is applied to the model without moderation effects to obtain weights to calculate the scores for the composites and latent variables. In the second step, the factor loadings are estimated by modifying the weights. In the final step, the structural coefficients are estimated using the method of moments estimator in combination with a correction for attenuation if latent variables are involved. Although PLSc can be used to study moderated mediation effects involving composites (Becker, Ringle, & Sarstedt, 2018; Belanche Gracia, Casaló Ariño, & Guinalíu Blasco, 2015; Henseler, 2021; Schuberth, 2019), it prevents researchers from taking full advantage of SEM’s capabilities, such as restricting model parameters or making use of SEM’s model evaluation criteria. While PLSc is implemented in the R package cSEM (Rademaker & Schuberth, 2020), researchers cannot use traditional SEM tools such as Mplus (Muthén & Muthén, 1998-2017) or the R package lavaan (Rosseel, 2012; Rosseel, Jorgensen, & De Wilde, 2025b).

**Fig. 2:**
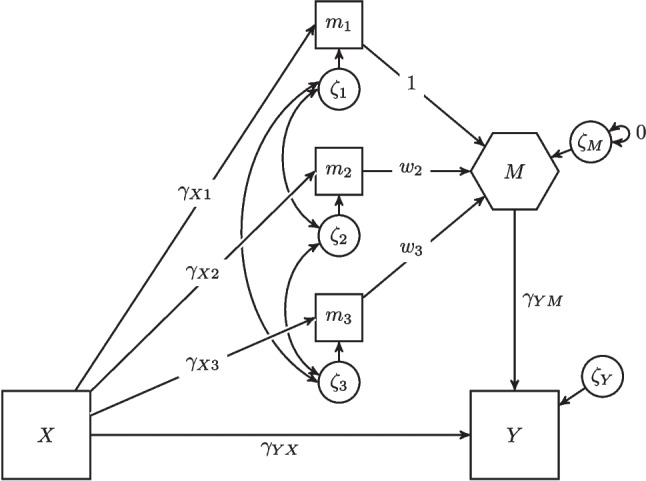
An example of the one-step approach with a composite as mediator variable. *Note*: The variables *X* and *Y* are observed variables, the variable *M* represents the composite formed by the three components $m_1$, $m_2$, and $m_3$

In addition to PLSc, several SEM approaches have been proposed for studying composites that may have the potential to analyze moderated mediation effects involving composites. For example, the two-step approach first creates composites and then uses these scores in the second step to estimate the relations between the composite and other variables in the model (see e.g., Schuberth, 2023). In contrast, the pseudo-indicator approach (Rose et al., 2019) can be used to model fixed-weight composites, including their observed variables in SEM. However, none of these approaches allows researchers to model composites with unknown weights. In the following section, we present the SEM approaches that allow for both fixed-weight composites and unknown-weight composites, i.e., the composite is modeled, and its weights can be fixed or freely estimated. We also discuss the suitability of these approaches for moderated mediation.

#### The one-step approach

In the *one-step approach*, a composite is specified as a latent variable in a causal-formative measurement model whose error variance is fixed to zero (e.g., Grace and Bollen, 2008). As the variance of the error term is fixed to zero, the latent variable is entirely composed of its observed variables, making it a composite. An example of a composite specified using the one-step approach is shown in Fig. 2. Following Grace and Bollen (2008), we draw composites as hexagons to distinguish them from latent and observed variables, which are usually drawn as ovals and rectangles, respectively.

The one-step approach allows for the specification of fixed-weight and unknown-weight composites in structural equation models. However, its flexibility in specifying composites is limited. For instance, it is not possible to specify covariances between composites because these covariances would need to be specified between the error terms of the latent variables, whose variances are fixed to zero (Schuberth, 2023). As a workaround, one can specify covariances between the observed variables that make up the composites. However, the literature offers conflicting suggestions in this regard (Grace & Bollen, 2008; MacCallum & Browne, 1993). Similarly, it is not possible to specify the effects of variables other than the components of the composite on the composite. In this case, the model would not be identified (MacCallum & Browne, 1993); consequently, composites can only be specified as predictor variables. With this in mind, the one-step approach cannot be used directly to specify a composite as a mediator, and thus cannot be used to specify a composite as a moderated mediator.

A potential way to address this issue is illustrated in Fig. 2. Rather than modeling the effect of the predictor variable *X* on the mediator *M* directly, the effect is modeled indirectly via the variables that make up the composite, i.e., $m_1$, $m_2$, and $m_3$. Subsequently, the observed variables become endogenous variables, and potential covariances between the observed variables are modeled via their error terms $\zeta _1$, $\zeta _2$, and $\zeta _3$. However, this way of specifying composites as mediators has a major disadvantage: It increases the number of model parameters, which complicates their interpretation. This problem is particularly prominent in the case of moderated mediation, when the effect of *X* on *M* is moderated by other variables (see Fig. 1b). Additionally, the model will not be identified if the composite *M* has no effects on other variables in the model.

#### The H–O specification

The discussed limitations of the one-step approach have been overcome by the recently proposed H–O specification (Henseler, 2021; Schuberth, 2023; Yu, Schuberth, & Henseler, 2023). In the H–O specification, not just one but as many composites as there are components are extracted from a set of observed variables.^1^ Furthermore, to enable flexible modeling of composites, the H–O specification expresses the relations between the composites and the observed variables $\boldsymbol{c}$ in terms of composite loadings $\boldsymbol{\Lambda }$ rather than weights $\boldsymbol{W}$:

1 $$\begin{aligned} \begin{pmatrix} C \\ \boldsymbol{\nu } \end{pmatrix} = \boldsymbol{W}' \boldsymbol{c} \qquad \Leftrightarrow \qquad \boldsymbol{c} = (\boldsymbol{W}')^{-1} \begin{pmatrix} C \\ \boldsymbol{\nu } \end{pmatrix} = \boldsymbol{\Lambda } \begin{pmatrix} C \\ \boldsymbol{\nu } \end{pmatrix}, \end{aligned}$$

where the matrix $\boldsymbol{W}$ contains in its columns the weights used to form the composite of interest *C* and the additional composites $\boldsymbol{\nu }$, called excrescent variables. The excrescent variables account for the variances and covariances between the components that cannot be explained by the composite of interest. The matrix $\boldsymbol{\Lambda }$ contains the composite loadings in its columns. Both composite loadings and weights are regression coefficients. Weights represent the contribution of components to the composite, while composite loadings can be interpreted as the composite’s influence on its components.

To ensure model identification, we use the identification rules of the refined H–O specification with free weights (Yu et al., 2023):^2^

- One composite loading per composite is fixed to one.
- Each excrescent variable is related to exactly two observed variables.
- Each observed variable loads on two excrescent variables at most.
- The excrescent variables are uncorrelated with the composites of interest.
- The composite of interest must be related to at least one other variable in the model in addition to its components.

A detailed discussion of how to achieve identification for the H–O specification is provided in Schuberth (2023) and Yu et al. (2023).

**Fig. 3:**
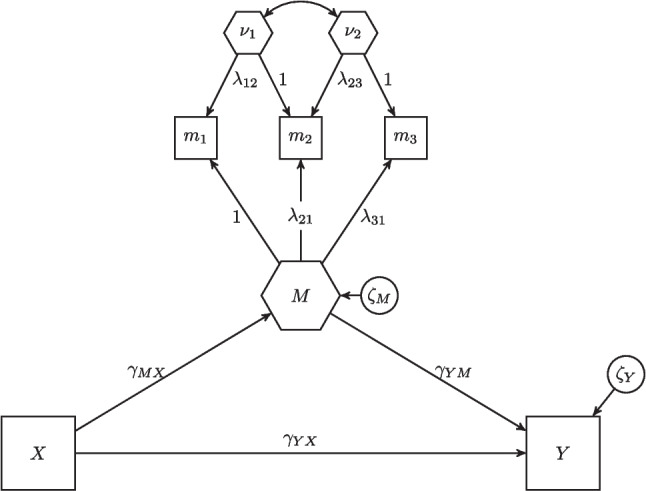
An example of a composite specified as mediator using the H–O specification

The H–O specification enables researchers to include composites as both predictor and outcome variables in a structural model. Therefore, it can be used directly to model mediation involving composites (Henseler & Schuberth, 2024). In addition, unlike the one-step approach, specifying composites as mediators does not increase the number of model parameters. Figure 3 illustrates how a composite can be modeled as a mediator using the H–O specification. The model consists of five observed variables: *X*, the predictor variable; *Y*, the outcome variable; and $m_1$, $m_2$, and $m_3$, the components. As explained previously, three composites are extracted from these components: the mediator *M*, and the excrescent variables $\nu _1$, and $\nu _2$. Composite loadings represent the relations between the composites and their components:

2 $$\begin{aligned} \begin{pmatrix} m_1\\ m_2 \\ m_3 \end{pmatrix} = \begin{pmatrix} 1 & \lambda _{12} & 0\\ \lambda _{21} & 1 & \lambda _{23}\\ \lambda _{31} & 0 & 1 \end{pmatrix} \begin{pmatrix} M \\ \nu _1 \\ \nu _2 \end{pmatrix} \end{aligned}$$

In addition to its flexibility in terms of model specification, the H–O specification can be used to model fixed-weight and unknown-weight composites (Henseler et al., 2025; Schuberth, Schamberger, Kemény, Henseler, 2025). Thus, it is a promising starting point for modeling moderated mediation effects with composites, as we will discuss in the next section.

## Modeling moderated mediation effects with composites: The composite moderated structural equations (CMS) approach

In this section, we introduce composite moderated structural equations (CMS), an extension of LMS for estimating moderated mediation involving composites. CMS combines the H–O specification described above with LMS. LMS is a full information maximum likelihood estimator for structural equation models that include moderation effects of latent variables (Klein & Moosbrugger, 2000; Moosbrugger, Schermelleh-Engel, & Klein, 1997). In the first step, the likelihood function of the observed variables is approximated. This likelihood function explicitly accounts for the non-normality of the observed variables induced by the latent variables’ moderation effects. In a second step, the likelihood function is maximized using an expectation maximization (EM) algorithm to obtain the parameter estimates. These estimates are asymptotically unbiased and efficient (Moosbrugger et al., 1997), making this the standard approach for nonlinear structural equation models against which other methods should be compared (cf. Dijkstra & Schermelleh-Engel, 2014; Ng & Chan, 2020). More details on LMS can be found in Klein and Moosbrugger (2000) and Kelava et al. (2011). For a comparison of LMS with existing approaches to moderation analysis in SEM with latent variables, see Brandt, Kelava, and Klein, (2014); Brandt, Umbach, Kelava, and Bollen (2020).

CMS blends LMS with the H–O specification, enabling its application to composite models. CMS is conceptualized as a three-step procedure. In the first step, the composite loadings $\boldsymbol{\Lambda }$ are estimated to compute the composite weights. For this purpose, we specify a model without moderation effects, allowing all latent variables and composites to freely covary, i.e., no constraints are imposed on the structural model. In principle, this means conducting a confirmatory composite/factor analysis (CCFA, Hubona, Schuberth, & Henseler, 2021), which is a combination of confirmatory factor analysis (CFA, Jöreskog, 1969) and confirmatory composite analysis (CCA, Schamberger et al., 2023; Schuberth, Henseler, & Dijkstra, 2018a). This approach is conceptually similar to the two-step approach known from SEM (Anderson & Gerbing, 1988). Classic SEM estimators such as the maximum likelihood estimator can be used to estimate the model parameters (Yu et al., 2023).

In the second step, scores are calculated for all composites of interest. This is done by inverting the estimated composite loading matrix to obtain the weight estimates that are used to calculate these scores:

3 $$\begin{aligned} \hat{\boldsymbol{W}} = \left( \hat{\boldsymbol{\Lambda }}'\right) ^{-1} \end{aligned}$$

Following the identification rules of the refined H–O specification, we can order the loading matrix in such a way that the first and last observed variables load on exactly one excrescent variable and the remaining $K-2$ observed variables load onto exactly two excrescent variables. Consequently, the estimated composite loading matrix takes the following form:

4 $$\begin{aligned} \hat{\boldsymbol{\Lambda }} = \begin{pmatrix} \hat{\lambda }_{11} & \hat{\lambda }_{12} & 0 & 0 & 0& \dots & 0\\ \hat{\lambda }_{21} & \hat{\lambda }_{22} & \hat{\lambda }_{23} & 0 & 0 & \dots & 0\\ \hat{\lambda }_{31} & 0 & \hat{\lambda }_{33} & \hat{\lambda }_{34} & 0 & \dots & 0\\ \hat{\lambda }_{41} & 0 & 0 & \hat{\lambda }_{44} & \hat{\lambda }_{45} & \dots & 0\\ \hat{\lambda }_{51} & 0 & 0 & 0 & \hat{\lambda }_{55} & \hat{\lambda }_{56} & \dots \\ \vdots & \vdots & \vdots & \vdots & \vdots & \ddots & \vdots \\ \hat{\lambda }_{K1} & 0 & 0 & 0 & 0 & \dots & \hat{\lambda }_{KK} \end{pmatrix}, \end{aligned}$$

where the first column contains the estimated composite loadings of the composite of interest. The remaining columns contain the composite loading estimates of the $K-1$ excrescent variables. The weights used to form the composites are obtained as the inverse of the estimated composite loading matrix, see Eq. 3. Consequently, the weights used to form the composite of interest, which equal the first column of $\hat{\boldsymbol{W}}$, are given as follows:

5 $$\begin{aligned} \frac{1}{|\hat{\boldsymbol{\Lambda }}|} \begin{pmatrix} |\text {diag}(\hat{\boldsymbol{\Lambda }})|/ \hat{\lambda }_{11}\\ -\hat{\lambda }_{12} \cdot |\text {diag}(\hat{\boldsymbol{\Lambda }})|/ (\hat{\lambda }_{11} \cdot \hat{\lambda }_{22}) \\ \hat{\lambda }_{12} \hat{\lambda }_{23} \cdot |\text {diag}(\hat{\boldsymbol{\Lambda }})|/ (\hat{\lambda }_{11} \cdot \hat{\lambda }_{22} \cdot \hat{\lambda }_{33}) \\ -\hat{\lambda }_{12} \hat{\lambda }_{23} \hat{\lambda }_{34} \cdot |\text {diag}(\hat{\boldsymbol{\Lambda }})|/ (\hat{\lambda }_{11} \cdot \hat{\lambda }_{22} \cdot \hat{\lambda }_{33} \cdot \hat{\lambda }_{44}) \\ \vdots \\ (-1)^{K-1}\hat{\lambda }_{12} \hat{\lambda }_{23} \hat{\lambda }_{34} \dots \hat{\lambda }_{KK} \end{pmatrix} \end{aligned}$$

where $|\hat{\boldsymbol{\Lambda }}|$ is the determinant of $\hat{\boldsymbol{\Lambda }}$, and |diag($\hat{\boldsymbol{\Lambda }}$)| is the product of the main diagonal elements of $\hat{\boldsymbol{\Lambda }}$. The composite weight estimates are used to create the desired scores by multiplying these weights by the respective components.

**Fig. 4:**
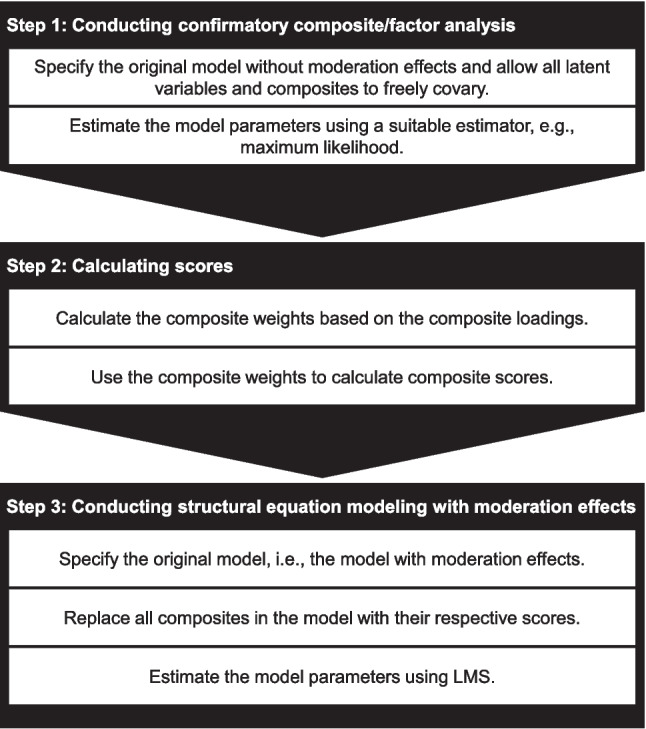
The three steps of CMS: A new approach to estimating structural equation models with composites involving moderated mediation

In the third step, we replace all the composites of the original model with their respective scores. Finally, we estimate the model parameters using LMS. Figure 4 illustrates the three steps of our CMS approach to estimating moderated mediation effects with composites.

## Monte Carlo simulation

We conduct a Monte Carlo simulation to demonstrate the performance of CMS. The purpose of this simulation study is twofold: (i) to demonstrate the performance of CMS in terms of bias and variability of its parameter estimates in finite samples, and (ii) to compare the finite-sample performance of CMS with that of PLSc. We deliberately chose PLSc because it is the only other approach that can handle moderated mediation models with composites and is implemented in the R package cSEM (Rademaker & Schuberth, 2020).

In our simulation study, we consider a population model with two latent variables and one composite: an exogenous latent variable *X*, a mediator composite *M*, and a latent outcome variable *Y*. Each of the two latent variables *X* and *Y* is measured by three observed variables:

6 $$\begin{aligned} x_1&\!=\! 0.7 \cdot X + \delta _{1}; \quad x_2 \!=\! 0.7 \cdot X + \delta _{2}; \quad x_3 = 0.7 \cdot X + \delta _{3};\end{aligned}$$

7 $$\begin{aligned} y_1&\!=\! 0.7 \cdot Y + \varepsilon _{1}; \quad y_2 \!=\! 0.8 \cdot Y + \varepsilon _{2}; \quad y_3 = 0.9 \cdot Y + \varepsilon _{3}, \end{aligned}$$

where the random measurement errors $\delta$ and $\varepsilon$ have a mean of 0, are mutually uncorrelated, and are uncorrelated with the latent variable *X*. The variances of the random measurement errors are set in such a way that the observed variables all have a unit variance.

To investigate the influence of heterogeneity in the composite weights on the results, we consider two weight sets to form the composite *M*:

- Weight Set 1: $M = 0.2\cdot m_1 + 0.4 \cdot m_2 + 0.6 \cdot m_3$, where $m_1$, $m_2$, and $m_3$ are correlated with 0.5.
- Weight Set 2: $$M = 0.4 \cdot m_1 + 0.5 \cdot m_2 + 0.5 \cdot m_3$$, where cor($$m_1, m_2) = 0.25$$, cor($$m_1, m_3) = 0.4$$, and cor($$m_2, m_3) = 0.16$$.

The variances of $m_i$, $i=1,2,3$ were set to 1 and their means to zero. In addition, the covariances between $m_1, m_2, m_3$, and the latent variable *X* were set to $0.4 \cdot \boldsymbol{\Sigma }_{mm} \boldsymbol{W}$, where $\boldsymbol{\Sigma }_{mm}$ displays the variance–covariance matrix of $m_1$, $m_2$, and $m_3$. Further, $m_1$, $m_2$, and $m_3$ are uncorrelated with the random measurement errors. Consequently, the composite fully accounts for the covariances between its components and other variables in the model.

**Fig. 5:**
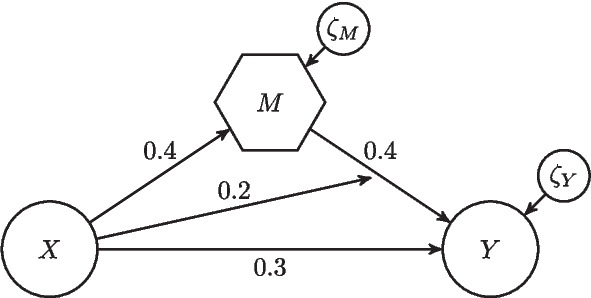
Illustration of the structural model for the Monte Carlo simulation

Figure 5 illustrates the structural model used in our Monte Carlo simulation. The equations of the structural model are given as follows:

8 $$\begin{aligned} M&= 0.4 \cdot X + \zeta _M \end{aligned}$$

9 $$\begin{aligned} Y&= 0.3 \cdot X + 0.4 \cdot M + 0.2(X\cdot M - \text {Cor}(X,M))+ \zeta _Y \\&= 0.3 \cdot X + 0.4 \cdot M + 0.2 \cdot X \cdot M + \zeta _Y - 0.2 \cdot \text {Cor}(X,M)\nonumber \\&= 0.46 \cdot X + 0.08 \cdot X^2 + (0.4 \cdot \zeta _M + 0.2 \cdot X \cdot \zeta _M +\zeta _Y) -0.08,\nonumber \end{aligned}$$

where the error terms $\zeta _M$ and $\zeta _Y$ have a mean of 0. Additionally, they are mutually uncorrelated and independent of *X*. They are also uncorrelated with the observed variables $m_1$, $m_2$, and $m_3$, as well as the random measurement errors. The latent variable *X* has a mean of zero and a unit variance, i.e., $\text {E}(X^2) = 1$. The variances of $\zeta _M$ and $\zeta _Y$ are chosen in such a way that *M* and *Y* have a unit variance. Moreover, the interaction term is mean-centered such that *Y* has a mean of zero. Consequently, *X*, *M*, and *Y* are standardized. The moderation effect is set to 0.2, which equals an effect size $f^2$ of approximately 0.07 for both weight sets, and thus represents a small effect (Cohen, 1992). The $R^2$ values are 0.12 and 0.16 for *M* and 0.38 and 0.39 for *Y*, depending on the weight set chosen. Further, and as shown in Eq. 9, when *X* moderates the effect of *M* on *Y*, it is equivalent to including a quadratic effect of *X* on *Y*.

**Table 1:** Share of inadmissible results for CMS and PLSc under the various simulation conditions

Weight set	Sample size	Inadmissible results for CMS	Inadmissible results for PLSc
1	250	6.34%	1.11%
1	500	1.96%	0.00%
1	1000	0.20%	0.00%
2	250	0.00%	1.38%
2	500	0.00%	0.00%
2	1000	0.00%	0.00%

We use the following procedure to simulate the data in the statistical environment R (R Core Team, 2023). First, we generate observations for the exogenous variables, i.e., *X*, $\zeta _Y$, $\delta _{1}$, $\delta _{2}$, $\delta _{3}$, $m_1$, $m_2$, $m_3$, $\varepsilon _{1}$, $\varepsilon _{2}$, and $\varepsilon _{3}$, from the multivariate normal distribution. For this purpose, we use their variance–covariance matrix and the mvrnorm function of the MASS package (Venables & Ripley, 2002). The mean vector of the exogenous variables is set to 0. Subsequently, we generate observations for the endogenous variables using the relations between the exogenous and endogenous variables as described in the population model (see Equations 6–9).

**Fig. 6:**
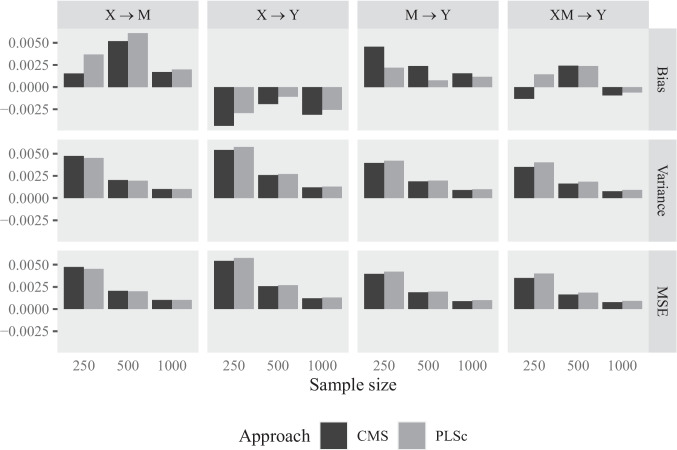
Bias, variance and MSE for CMS and PLSc in case of Weight set 2

To assess the finite sample behavior of CMS, we vary the sample sizes from 250, 500 to 1000 observations. Consequently, we apply the two approaches, i.e., CMS and PLSc, to six conditions: three sample sizes (*N* = 250, 500, 1000), and two different weight sets. For each condition, we draw 500 samples. Each sample is analyzed using CMS and PLSc. In case PLSc or CMS produced an inadmissible solution, the sample was omitted from the analysis, and a completely new sample was drawn.^3^ Thus, the results of the two approaches are based on the same datasets, and the results for each condition are based on 500 admissible solutions.

To estimate the model parameters using CMS, we follow the procedure described above. First, we estimate a CCFA model, in which *X*, *M*, and *Y* are related to their observed variables and are allowed to freely covary. For this purpose, we use the R package lavaan. Second, we build a score for the composite *M*. To do so, we calculate the composite weights using the estimated composite loadings from the first step. Subsequently, we multiply these composite weights by the respective observed variables $m_1$, $m_2$, and $m_3$. Additionally, we mean-center the observed variables so that no intercepts need to be estimated for the observed variables; only an intercept for *Y* is included (see Eq. 9).

We estimate the model that involves the moderation effect by LMS using the R package modsem (Slupphaug et al., 2025). To do so, we replace *M*, including its components, with its score $\hat{M}$. Finally, we standardize the CMS estimates to ensure a fair comparison with PLSc. To obtain the PLSc estimate, we used the R package cSEM (Rademaker & Schuberth, 2020).

Table 1 illustrates the shares of inadmissible results for CMS and PLSc under the different simulation conditions. Both approaches yield a small share of inadmissible results, where CMS shows a larger share of inadmissible results for the first weight set. Note that the inadmissible solutions in CMS mostly occurred during the first step in all considered conditions. As sample sizes increase, the proportion of inadmissible solutions approaches zero for all conditions.

Figure 6 displays the results of our Monte Carlo simulation. Since the results are very similar for the two considered weight sets, we only report the results for Weight Set 2.^4^ The bars represent the estimated bias, variance, and mean squared error (MSE; see e.g., Casella & Berger, 2024) for the different conditions for the four structural parameters:

$$\begin{aligned} \widehat{\text {Bias}}(\hat{\theta })&= \frac{1}{500} \sum _{i = 1}^{500} (\hat{\theta } - \theta ) \\ \widehat{\text {Var}}(\hat{\theta })&= \frac{1}{499} \sum _{i = 1}^{500} (\hat{\theta } - \theta )^2\\ \widehat{\text {MSE}}(\hat{\theta })&= \widehat{\text {Bias}}(\hat{\theta })^2 + \widehat{\text {Var}}(\hat{\theta }) \end{aligned}$$

Figure 6 shows that, on average, CMS leads to estimates that are close to their population counterparts for all simulation conditions. Additionally, as the sample size increases, the MSE and the variance decrease, indicating that the parameter estimates become more precise with an increasing sample size. This is in line with the asymptotic properties of LMS estimates. A comparison of the results with those of PLSc shows that neither method has a clear advantage in terms of bias or variance, and MSE.

Finally, we evaluate the power of both CMS and PLSc to detect the moderation effect using a significance level of 5%. For CMS, we used the standard errors obtained by LMS, whereas for PLSc, we used bootstrap standard errors to calculate the respective z-test statistics. As shown in Table 2, both CMS and PLSc show a power greater than the desired level of 80% in all conditions (Cohen, 1988). As expected, the power increased for both approaches with an increasing sample size. Finally, the power of CMS was higher than the power of PLSc for all considered conditions.^5^

**Table 2:** Power of the *z*-test used to test the moderation effect (5% significance level)

Weight set	Sample size	CMS	PLSc
1	250	89.40%	84.20%
1	500	99.80%	99.20%
1	1000	100.00%	100.00%
2	250	91.40%	87.60%
2	500	99.60%	99.00%
2	1000	100.00%	100.00%

## Discussion

Composites have been established as a useful type of variable in SEM. For example, they can be used to model collections of heterogeneous causes (Grace & Bollen, 2008) or formative constructs (e.g., Fornell, 1982). However, approaches that can be used to estimate moderated mediation effects involving composites are limited. Candidates such as the two-step approach and the pseudo-indicator approach are limited to fixed-weight composites; therefore, they cannot be used to study moderated mediation effects involving unknown-weight composites. The one-step approach can be used to study unknown-weight composites; however, in its original form, it is limited to composites that are unaffected by other variables. Consequently, it can only be used to study moderated mediation effects in which the composites are neither mediators nor outcome variables. As one potential solution, we suggest specifying the effects of a predictor variable on the components rather than on the composite directly. However, this way of studying composites is very generous in terms of model parameters, which can be problematic, particularly in the case of moderated mediation. Moreover, approaches that have evolved outside the realm of SEM, such as PLSc, prevent researchers from benefiting from the advantages of SEM. To address these limitations, we introduce CMS to estimate moderated mediation models involving composites. CMS combines LMS, a full information maximum likelihood estimator to estimate the model parameters of structural equation models involving latent variables, with the refined H–O specification for composites. This combination enables researchers to study moderated mediation effects involving composites with great flexibility.

To evaluate the performance of CMS, we performed a Monte Carlo simulation using a model where the effect of the predictor *X* on the outcome *Y* is mediated by a composite *M* and the effect of *M* on *Y* is moderated by *X*. Our results show that, on average, the parameter estimates produced by CMS are close to their population counterparts. Furthermore, the parameter estimates become more precise as the sample size increases. We also compared the performance of CMS with that of PLSc and found that the two approaches produced similar results. In particular, the bias, variance, and MSE of the parameter estimates produced by CMS were similar to those produced by PLSc. However, when testing the moderation effect, CMS outperformed PLSc in samples with 250 observations due to its higher statistical power.

Naturally, Monte Carlo simulations, including ours, are limited by their design. For example, in our simulation, we estimated only one type of moderated mediation effect. While we do not anticipate any differences to our main conclusion regarding the performance of CMS, future research could examine CMS in situations that encompass other types of moderated mediation (see Fig. 1). Moreover, we generated observations of exogenous variables from a multivariate normal distribution. As highlighted in the literature, LMS and thus CMS rely on the normality assumption of the exogenous variables (e.g., Klein & Moosbrugger, 2000; Lonati, Rönkkö, & Antonakis, 2024). Therefore, future research should examine the negative effects of violating this assumption of CMS (as has been done for LMS, e.g., Brandt et al., 2020). Additionally, we have only considered the case where the composite is a mediator variable. While a standard LMS can be applied if the composite is a predictor variable and both the mediator and the outcome variable are latent variables, CMS needs to be applied if the composite is a mediator or an outcome variable. Therefore, future research should examine the performance of CMS if a composite is (additionally) an outcome variable. Finally, our simulation study did not consider misspecified models. It is well known that misspecified models can result in biased parameter estimates. Consequently, future research should investigate the impact of model misspecifications on CMS.

There are several advantages to using CMS: First, it enables researchers to use software in which LMS is implemented, such as the commercial software Mplus or the open-source R package modsem. Second, our approach is based on LMS, which has well-known asymptotic properties. Finally, our approach uses the H–O specification, which allows researchers to utilize its extensions, such as modeling fixed-weight composites (Henseler et al., 2025; Schuberth et al., 2025). However, our approach also has certain disadvantages. Since CMS is based on LMS, it inherits all of LMS’s limitations, such as its limitations when testing the overall model fit (see e.g., Gerhard, Büchner, Klein, & Schermelleh-Engel, 2017). As with LMS, these limitations can be addressed using a two-step test procedure (Klein & Moosbrugger, 2000; Maslowsky, Jager, & Hemken, 2014). First, the likelihood ratio test statistic of the model without moderation effects is used. Then, the significance of the interaction effect can be evaluated. Future research should evaluate this procedure for CMS. Moreover, using CMS involves a three-step procedure (see Fig. 4), which may be more complex than using a one-step approach. In analogy to the structural after measurement (SAM, Rosseel et al., 2025a; Rosseel & Loh, 2024), CMS can be conceived as a structural after synthesis (SAS) approach. Furthermore, in the final step of CMS, the unknown-weight composites are no longer modeled, but replaced by their scores, thus ignoring the uncertainty in the weight estimates. This can lead to biased standard errors of the parameter estimates, as we, for example, know from factor score regression with fixed error variances (Oberski & Satorra, 2013; Rosseel & Loh, 2024). Future research should evaluate the effect of ignoring the uncertainty in the weight estimates on the standard errors and suggest ways to account for it. One way to address this issue is to use bootstrap to quantify the uncertainty in the parameter estimates (Efron & Tibshirani, 1994), as done in PLSc (Aguirre-Urreta & Rönkkö, 2018). Moreover, CMS assumes that the components that make up a composite are free from measurement error. However, measurement error is often present in variables (Buonaccorsi, 2010). Therefore, future research should focus on ways to account for measurement error in the components when using CMS.

## Conclusion

CMS is a promising starting point for estimating moderated mediation and other nonlinear models involving composites, which expands the applicability of SEM for studying composites.

## Supplementary Information

Below is the link to the electronic supplementary material.

Supplementary file 1 (pdf 70 KB)

## Data Availability

The results of the Monte Carlo simulation can be downloaded here: https://osf.io/4t9se.

## Footnotes

1 It is assumed that the variables that make up a composite are not perfectly linearly dependent.

2 In the H–O specification, the composites fully span the space of the components. For this purpose, various other specifications are available, see, e.g., Schuberth (2023); Henseler, Yu, Schamberger, Hancock, and Schuberth (2025)

3 The R code for conducting the Monte Carlo simulation and the results can be downloaded via the following URL: https://osf.io/4t9se.

4 A table with the complete results is provided in the Supplementary Material.

5 We have rerun the simulation study and set the moderation effect in the population to zero (XM$\rightarrow$ Y). The corresponding results showed that the type I error rates were close to 5% under all conditions considered.

## Code availability

The code used for the analysis can be downloaded here: https://osf.io/4t9se.

## Declarations

### Open Practices Statement

The analysis in this article is based on simulated data. The code used for these simulations and the results of the Monte Carlo simulation are available via: https://osf.io/4t9se.
