# Supplementary material for: Moderated mediation with composites: The composite moderated structural equations approach
Source: Behav Res Methods. 2026 Apr 6;58(4):104. doi: 10.3758/s13428-025-02930-w (PMC13053372; doi:10.3758/s13428-025-02930-w)
Supplement: Supplementary file 1 — (pdf 70 KB) [file 13428_2025_2930_MOESM1_ESM.pdf]

Supplementary Material to: Moderated Mediation with Composites: The Composite Moderated Structural Equations Approach

Tamara Schamberger[1], Florian Schuberth[2], and Jörg Henseler[2,3]

[1]Bielefeld University, Faculty of Business Administration and Management, Bielefeld, 33615, Germany
[2]University of Twente, Department of Design, Production and Management, Enschede, 7500 AE, The Netherlands
[3]Universidade Nova de Lisboa, Nova Information Management School, Campus de Campolide, 1070-312 Lisboa, Portugal

Table 3: Bias and Standard Deviations for the Factor Loadings and Composite Weights for the Monte Carlo Simulation

Weight Set	Sample Size	Estimator		$X \rightarrow x_1$	$X \rightarrow x_2$	$X \rightarrow x_3$	$Y \rightarrow y_1$	$Y \rightarrow y_2$	$Y \rightarrow y_3$	$m_1 \rightarrow M$	$m_2 \rightarrow M$	$m_3 \rightarrow M$
1	250	CMS	Bias	-0.002	0.001	-0.002	-0.001	-0.001	-0.001	0.018	-0.010	-0.013
			SD	0.048	0.046	0.052	0.039	0.032	0.024	0.111	0.109	0.107
		PLSc	Bias	-0.005	-0.004	0.000	-0.002	-0.001	-0.002	0.015	-0.010	-0.012
			SD	0.076	0.075	0.077	0.064	0.050	0.042	0.113	0.111	0.111
	500	CMS	Bias	0.001	-0.003	-0.002	0.000	0.000	0.000	0.002	-0.013	0.003
			SD	0.032	0.035	0.033	0.028	0.022	0.019	0.080	0.091	0.085
		PLSc	Bias	0.002	-0.004	-0.005	0.000	-0.001	0.000	0.002	-0.013	0.003
			SD	0.053	0.059	0.054	0.046	0.036	0.031	0.082	0.092	0.086
	1000	CMS	Bias	-0.001	0.000	0.000	0.001	0.000	0.000	-0.002	-0.002	-0.001
			SD	0.025	0.025	0.023	0.018	0.015	0.013	0.062	0.062	0.059
		PLSc	Bias	-0.002	-0.001	0.001	0.002	0.000	-0.002	-0.002	-0.002	-0.001
			SD	0.039	0.039	0.038	0.030	0.026	0.022	0.064	0.063	0.060
2	250	CMS	Bias	0.002	0.000	0.000	-0.001	-0.002	-0.001	-0.015	0.005	-0.005
			SD	0.049	0.051	0.049	0.039	0.031	0.026	0.112	0.099	0.108
		PLSc	Bias	-0.002	-0.001	-0.002	0.002	-0.005	-0.003	-0.015	0.005	-0.005
			SD	0.077	0.077	0.076	0.062	0.053	0.041	0.112	0.099	0.108
	500	CMS	Bias	0.000	-0.001	0.001	-0.001	0.000	0.000	-0.008	0.004	-0.002
			SD	0.035	0.035	0.033	0.027	0.022	0.018	0.077	0.068	0.074
		PLSc	Bias	-0.002	0.000	-0.001	0.001	-0.001	-0.003	-0.009	0.004	-0.002
			SD	0.054	0.055	0.055	0.044	0.037	0.032	0.078	0.069	0.076
	1000	CMS	Bias	-0.001	0.001	-0.001	-0.001	-0.001	0.000	0.004	-0.005	-0.004
			SD	0.024	0.024	0.024	0.020	0.015	0.013	0.057	0.050	0.052
		PLSc	Bias	-0.002	0.001	-0.001	0.000	-0.001	-0.001	0.004	-0.004	-0.005
			SD	0.039	0.036	0.039	0.031	0.025	0.023	0.058	0.051	0.052

Table 4: Bias and Standard Deviations for the Path Coefficient Estimates for the Monte Carlo Simulation

Weight Set	Sample Size	Estimator	Result	$X \to M$	$X \to Y$	$M \to Y$	$XM \to Y$
1	250	CMS	Bias	-0.003	-0.007	0.014	-0.009
			SD	0.065	0.069	0.061	0.061
		PLSc	Bias	-0.001	-0.006	0.010	-0.007
			SD	0.065	0.073	0.064	0.066
	500	CMS	Bias	0.002	-0.002	0.004	0.000
			SD	0.045	0.051	0.044	0.040
		PLSc	Bias	0.002	-0.002	0.003	0.001
			SD	0.045	0.053	0.045	0.044
	1000	CMS	Bias	0.003	0.000	0.002	-0.001
			SD	0.031	0.036	0.032	0.030
		PLSc	Bias	0.003	0.000	0.002	0.000
			SD	0.030	0.038	0.034	0.032
2	250	CMS	Bias	0.002	-0.004	0.005	-0.001
			SD	0.069	0.074	0.063	0.059
		PLSc	Bias	0.004	-0.003	0.002	0.001
			SD	0.067	0.076	0.065	0.063
	500	CMS	Bias	0.005	-0.002	0.002	0.002
			SD	0.045	0.051	0.043	0.040
		PLSc	Bias	0.006	-0.001	0.001	0.002
			SD	0.044	0.052	0.044	0.043
	1000	CMS	Bias	0.002	-0.003	0.002	-0.001
			SD	0.032	0.035	0.030	0.028
		PLSc	Bias	0.002	-0.003	0.001	-0.001
			SD	0.032	0.036	0.032	0.030
